# Sympathetic and parasympathetic central autonomic networks

**DOI:** 10.1162/imag_a_00094

**Published:** 2024-02-28

**Authors:** Gaetano Valenza, Francesco Di Ciò, Nicola Toschi, Riccardo Barbieri

**Affiliations:** Neurocardiovascular Intelligence Lab, Bioengineering and Robotics Research Centre “E. Piaggio”, University of Pisa, Pisa, Italy; Department of Information Engineering, University of Pisa, Pisa, Italy; Department of Biomedicine and Prevention, University of Rome “Tor Vergata”, Rome, Italy; Athinoula A. Martinos Center for Biomedical Imaging, Department of Radiology, Massachusetts General Hospital, Harvard Medical School, Charlestown, MA, United States; Department of Electronics, Informatics and Bioengineering, Politecnico di Milano, Milano, Italy

**Keywords:** central autonomic network, sympathetic activity, parasympathetic activity, heart rate variability, fMRI, brain-heart axis

## Abstract

The central-autonomic network (CAN) comprises brain regions that are functionally linked to the activity of peripheral autonomic nerves. While parasympathetic CAN (i.e., the CAN projecting onto parasympathetic branches) has recently been investigated and is known to be involved in neurological and neuropsychiatric disorders, sympathetic CAN (i.e., the CAN projecting onto sympathetic nerves) has not been fully characterized. Using functional magnetic resonance imaging (fMRI) data from the Human Connectome Project in conjunction with heartbeat dynamics and its orthonormal autoregressive descriptors as a proxy for sympathetic activity estimation, namely, the sympathetic activity index (SAI), we uncover brain regions belonging to the sympathetic CAN at rest. We uncover a widespread CAN comprising both cortical (in all lobes) and subcortical areas, including the cerebellum and brainstem, which is functionally linked to sympathetic activity and overlaps with brain regions driving parasympathetic activity. These findings may constitute fundamental knowledge linking brain and bodily dynamics, including the link between neurological and psychiatric disorders and autonomic dysfunctions.

## 1 Introduction

The central autonomic network (CAN) is a group of brain regions that are functionally connected and linked to the autonomic nervous system (ANS), which are essential for the regulation and monitoring of cardiovascular function (Beissner et al., 2013; Benarroch, 2020; Valenza, Passamonti, et al., 2020; Valenza et al., 2019). The discovery of the CAN was first made by Benarroch (Benarroch, 1993) and it primarily consists of cortical (medial prefrontal cortex (mPFC), cingulate cortex (CC), insula, etc.), subcortical (amygdala, hypothalamus, thalamus), and brainstem regions (periaqueductal gray matter of the midbrain (PAG), and several nuclei in the medulla oblongata and pons) (Benarroch, 1993, 2012, 2020; Macefield & Henderson, 2019; Mulcahy et al., 2019; Saper, 2003). Each component in this network plays a distinct role in supporting the complex process of autonomic regulation under various physiological circumstances. The insula and cingulate cortex are part of the salience network and their activity while at rest is related to the activity of sympathetic muscle fibers (Benarroch, 2020; Macefield & Henderson, 2019). Together with the amygdala, mPFC, and frontal cortices, these cortical regions are crucial in regulating homeostatic-interoceptive functions that involve autonomic dynamics (Benarroch, 2020; M. Nagai et al., 2010; Patron et al., 2019; Pollatos et al., 2007; Valenza, Passamonti, et al., 2020). This leads to the theory that the brain’s visceromotor and viscerosensory representations form the foundation for emotion perception (Craig, 2002, 2009; M. Nagai et al., 2010).

Studies in the recent past have employed heart rate variability (HRV) series in association with neuroimaging methods to examine the functional neuroanatomy of the central autonomic network (CAN) (Beissner et al., 2013; Quadt et al., 2022). Typically, the arousal level of the participants is altered through affective, cognitive, or somatosensory/motor stimuli while monitoring cardiac activity via photoplethysmography or electrocardiogram (Goswami et al., 2011; Matsunaga et al., 2009; Nugent et al., 2011). Matsunaga et al. for instance, found a correlation between HF-HRV and the response of the insular cortex to positive emotions (Matsunaga et al., 2009). The use of sensory and motor activities has also been explored in order to understand the representation of somatosensory inputs within the CAN, with the ventromedial prefrontal cortex and subgenual cingulate cortex found to be involved in parasympathetic regulation (Goswami et al., 2011). Additionally, Nugent et al. noted distinct connections between HRV indices and orbitofrontal brain metabolism in depression patients as compared to controls (Nugent et al., 2011). Moreover, there are several studies that have investigated the link between HF-HRV fluctuations and cortical/subcortical fMRI signals (Chang et al., 2013; Napadow et al., 2008; Nikolaou et al., 2016; Sclocco, Beissner, et al., 2016; Sclocco, Kim, et al., 2016).

We recently used a technique for estimating heartbeat dynamics in real time (Valenza et al., 2018b) combined with resting-state functional MRI (rs-fMRI) data to describe the neural correlates of parasympathetic outflow and heartbeat dynamics (Valenza, Passamonti, et al., 2020; Valenza et al., 2019). Our findings indicate that the CAN is associated with a broad network of brain regions accounting for the nuanced and dynamic variations in CAN function that occur during rest compared to more polarizing task-based experiments (Reisert et al., 2021). It was interesting to observe a consistent negative relationship between vagal autonomic control and CAN activity. From a structural standpoint, recent tractography research has shown that the CAN extends to both cortical and subcortical regions of the brain (Reisert et al., 2021). The amygdala, a key cortical-subcortical pathway, along with various cortical regions such as the ventromedial, orbitofrontal, lingual, and medial prefrontal cortices, interact with one another to regulate brainstem activity, which, in turn, directly influences essential bodily functions (Lane, 2008; Lindquist et al., 2009; Thayer et al., 2012).

In the medulla, nuclei such as the parasympathetically active nucleus ambiguous and sympathetically active rostral ventrolateral and rostral ventromedial medulla receive direct inputs from higher regions, including the insula, PAG, and NTS (Macefield & Henderson, 2019; Nakamura & Morrison, 2022). These nuclei then project to either parasympathetic (vagal) or sympathetic preganglionic neurons (Silvani et al., 2016; Ter Horst & Postema, 1997; Verberne, 2011). The hypothalamus, with its multiple subregions, is instrumental in maintaining homeostasis and managing stress by coordinating a range of neuroendocrine, behavioral, and autonomic responses (Cechetto & Saper, 1988). The medulla integrates peripheral information, especially related to respiration, and connects to higher regions of the CAN through numerous nuclei, constituting the cardiorespiratory network (Richter et al., 1991).

The CAN has crucial implications for the development and advancement of various disorders. Disturbances in the brain-heart interaction and the subsequent changes in communication between the ANS and CNS (Singh et al., 2019; Thayer et al., 2012; Wang et al., 2020) have been linked to neurological injury (Riching et al., 2020; Tahsili-Fahadan & Geocadin, 2017), mood disorders, schizophrenia, anxiety (Catrambone et al., 2021; Sanchez-Gonzalez et al., 2014; Seligowski et al., 2022; Tumati et al., 2021; Yang & Tsai, 2013), chronic and acute stress conditions (Lamotte et al., 2021), epilepsy (Calandra-Buonaura et al., 2012), insomnia (Jiang et al., 2015), and Parkinsonism (Valenza et al., 2016). Additionally, vagal nerve stimulation has been proposed as a treatment for epilepsy and depression (Rossi et al., 2016), while biofeedback based on cardiovascular data has proven to be effective in regulating negative emotions and psychological symptoms (Karavidas et al., 2007).

Assessing ANS activity non-invasively often involves analyzing heartbeat information. Finger photoplethysmography (PPG) signals have frequently been utilized to determine cardiovascular variability in an fMRI design, which is then processed using spectral paradigms. Accurate assessment of cardio-vagal activity is obtained by calculating the spectral power of heartbeat variability in the high-frequency band (HF: 0.14-0.40 Hz) (Task Force of the European Society of Cardiology, 1996). Although spectral analysis has made it possible to study the functional brain correlation of vagal activity (parasympathetic CAN), this technique has yet to help us reveal cerebral correlation of sympathetic autonomic outflow (sympathetic CAN). Indeed, measuring time-varying sympathetic activity non-invasively is quite challenging. The spectral paradigm cannot effectively separate sympathetic activity as the heartbeat dynamics amplitude modulation below 0.14 Hz is influenced by both vagal and sympathetic oscillations, as well as baroreflex activity (Reyes del Paso et al., 2013; Valenza et al., 2018a). While previous attempts at studying the sympathetic Central Autonomic Network (CAN) through non-invasive and undirected measurements of sympathetic activities have met with limited success, Macefield and Henderson (2010) have made significant strides investigating this system. Their innovative approach involves the integration of Muscle Sympathetic Nerve Activity (MSNA) signals—sourced from the common peroneal nerve—with BOLD signals derived from fMRI. Their research revealed a correlation between the magnitude of nerve signals and the intensity of BOLD signals in specific medulla structures. Notably, they observed an increase in signal intensity within the rostral VLM region and decreases in regions linked with the NTS and caudal VLM. These observations underscore the critical involvement of these regions in the regulation of MSNA. Building on this methodology in a later study (James et al., 2013), they uncovered a positive correlation between MSNA and BOLD signals in several other cerebral regions, including the left dorsomedial hypothalamus (DMH), bilateral ventromedial hypothalamus (VMH), left insula, bilateral dorsolateral prefrontal cortex, bilateral posterior cingulate cortex (PCC), and bilateral precuneus (James et al., 2013; Macefield & Henderson, 2016, 2019).

In this study, we sought to address the difficulties in measuring sympathetic activity noninvasively using the time-resolved sympathetic activity index (SAI) (Valenza et al., 2018a, 2018b) to examine functional brain correlations with sympathetic activity at rest (sympathetic CAN). In addition, the parasympathetic activity index (PAI) is utilized to describe the parasympathetic CAN and compared to previous studies that utilized the spectral paradigm (Chang et al., 2013; Napadow et al., 2008; Nikolaou et al., 2016; Sclocco, Beissner, et al., 2016; Sclocco, Kim, et al., 2016; Valenza, Passamonti, et al., 2020; Valenza et al., 2019). The SAI and PAI are calculated noninvasively from heartbeat dynamics and are defined through a combination of disentangling coefficients and orthonormal expansion of autoregressive terms using Laguerre functions (Valenza et al., 2018a, 2018b). The combination of SAI and PAI indices with resting-state fMRI data sourced from the Human Connectome Project (HCP) (www.humanconnectome.org/) (McNab et al., 2013) provides higher temporal resolution compared to many resting-state fMRI studies and allows for the utilization of a large volume of data from young, healthy volunteers collected repeatedly.

## 2 Methods

### 2.1 Participants

This study utilized datasets selected from the Human Connectome Project’s “100-unrelated participants” data release (HCP U100, 1200 data release). The chosen sample comprised young and healthy adults aged between 22 and 36, who were free from any medical or neuropsychiatric conditions such as hypertension, alcoholism, anxiety disorders, or depressive disorders. Demographic information is presented in Table 1, while further details on subject inclusion and exclusion criteria can be found in the Supplementary Materials.

**Table 1.** Demographics of the sample population included in this study.

Age (years)	28.82 ± 3.37
Education (years)	15.03 ± 1.73
Height (centimeters)	170 ± 10
Weight (kilograms)	78.03 ± 39.92
Body mass index	26.76 ± 4.61
Systolic blood pressure (mmHg)	127.38 ± 13.86
Diastolic blood pressure (mmHg)	79.35 ± 9.14
Conduct problems during childhood (Number)	0.71 ± 1.06
Panic disorder symptoms (Number)	0.15 ± 0.36
Depressive symptoms (Number)	1.35 ± 1.15
Cigarettes per week (Number)	7.21 ± 21.20
Drinks per week (Number)	4.47 ± 5.00
Handedness (%)	62.35 ± 54.73
Ethnicity (%)	85.29% white;5.88% Unknown or Not Reported;5.88% Black or African American;2.94% More than one
Race (%)	79.41% Not Hispanic/Latino;20.59% Hispanic/Latino
Male (%)	47.06%

### 2.2 MRI and physiological data acquisition

The scanning and preprocessing methods are thoroughly described in the Human Connectome Project release handbook (www.humanconnectome.org). The participants underwent scans at Washington University in St. Louis using a Siemens 3 T Connectome Skyra scanner. The scans were conducted in two separate sessions, each of which included two resting state fMRI runs. Participants were instructed to keep their eyes open and focused on a crosshair displayed on a dark background in a dimly lit room. The acquisitions were performed in oblique axial orientation, alternating between right-to-left (RL) phase encoding in one run and left-to-right (LR) phase encoding in the other.

Functional MRI data was acquired using gradient-echo echo-planar imaging (EPI) with the following specifications: TR = 720 ms, TE = 33.1 ms, flip angle = 52 deg, FOV = 208 × 180 mm, matrix = 104 × 90, 72 slices, 2.0 mm isotropic voxel size, multiband factor = 8, echo spacing = 0.58 ms, and BW = 2290 Hz/Px. Each run consisted of 1200 volumes, resulting in a total acquisition time of approximately 15 minutes.

For preprocessing and data registration, a T1-weighted structural volume (3D MPRAGE, TR = 2400 ms, TE = 2.14 ms, TI = 1000 ms, flip angle = 8 deg, FOV = 224 × 224 mm, 0.7 mm isotropic voxel size, BW = 210 Hz/Px, multiband factor = 2) and spin echo field maps were generated using both RL and LR phase encoding methods. Cardiac data were acquired simultaneously with the fMRI data using a Siemens pulse oximeter attached to a participant’s finger and a respiratory belt. The data were sampled at 400 Hz. To guarantee the accuracy of the HRV indices, the cardiac data from each participant was examined by a skilled observer for factors such as signal-to-noise ratio, presence of ectopic beats, missing data, or other artifacts that could impact the HRV analysis. The study only included participants with usable datasets from the same session to reduce intersession bias, resulting in a final dataset of 34 full participants with two usable runs acquired on the same day. The final participant list and individual quality ratings of the cardiac recordings are described in the Supplementary Materials.

### 2.3 Data preprocessing

The preprocessing of the 1200-volume rs-fMRI data for each run was carried out by the HAC consortium using the FSL software (Jenkinson et al., 2012) as per the version 3.1 of the HCP pipeline, designed to maximize the utilization of HCP data quality (Glasser et al., 2013). A unique boundary-based registration algorithm was employed for each scanning day, including correction of distortions, registration into a standard space, correction of gradient distortions, motion correction using FLIRT, field map preprocessing with a spin echo field map using TOPUP, resampling into MNI space with all transforms through one-step spline method, normalization of intensity, and removal of bias fields. The ICA+ FIX tool (Salimi-Khorshidi et al., 2014), trained specifically for HPC data, was used to eliminate measurement noise-related data artifacts, excess motion, and physiological artifacts such as heartbeats and respiration.

### 2.4 Instantaneous estimation cardiovascular sympathetic/parasympathetic activity indices(SAI and PAI)

The proposed SAI-PAI approach is grounded in the understanding that the LF rhythm (centered at 0.1 Hz) in Heart Rate Variability (HRV) primarily originates from arterial baroreflex modulation and is significantly influenced by vasomotor noise. This noise is amplified by the resonance in the baroreflex loop around 0.1 Hz (Valenza et al., 2018a, 2018b). Past studies have associated the LF component of HRV’s power spectrum with sympathetic activity due to alterations in sympathetic gains. However, recent evidence suggests changes below 0.15 Hz can be directed by both cardiac vagal and sympathetic activity. The foundation of the SAI-PAI approach comes from recognizing that cholinergic and adrenergic drives exhibit distinct temporal dynamics, which overlap partially in the frequency domain. Instead of utilizing base functions defined in narrow frequency ranges, the SAI-PAI approach applies a weighted sum or subtraction of primitives that encompass the full frequency domain. These primitives, derived from discrete-time orthonormal Laguerre bases with different phase spectra and identical magnitude for a given α, effectively decompose heartbeat variability attributed to ANS activity. This breakdown assists in isolating the unique contributions of each autonomic branch, thereby enabling a more comprehensive understanding of the complex interactions between sympathetic and parasympathetic systems in HRV analysis.

In detail, the PPG signal was used to obtain the heart period (HP) series by automatically identifying the local maxima corresponding to heartbeat events. Within the context of the inhomogeneous point-process framework, we estimate the probability density function (PDF) characterizing each individual heartbeat event by considering past HP intervals. Subsequently, the heartbeat dynamics series undergoes convolution with Laguerre filters. The mean of the PDF associated with each heartbeat, modeled as an Inverse-Gaussian function, is then modeled as a linear combination of Laguerre coefficients. Through a subsequent linear combination of these Laguerre coefficients, we derive an estimation of the SAI and PAI (Valenza et al., 2018a, 2018b). Since the PDF associated with every heartbeat is defined in the continuous time *t*, the Laguerre coefficients *g(t)* are defined in the continuous time as well, allowing for a dynamic (i.e., instantaneous) assessment of SAI and PAI. The two indices are expressed in arbitrary units, with lower bound value set at zero.

In this study, the heart period (HP) series derived from the PPG signal was adjusted for physiological and algorithmic artifacts using the automated methods (Citi et al., 2012)

More in detail, let us consider an observation in time with length *T*, with t∈(0,T], given an ordered set of *K* timing of heartbeat events ${\left\{u_{k}\right\}}_{k=1}^{K}$ with $0\leq u_{1}<\ldots <u_{k}$$<u_{k+1}<\ldots <u_{K}\leq T$, and $HP_{k}=u_{k}-u_{k-1}>0$ the $k^{th}$ heartbeat period, the probability function describing the wait time until the k^th^ heartbeat period follows an inverse-Gaussian function:

$$f\left(t|{ℋ}_{t},\xi \left(t\right)\right)=\sqrt{\frac{\xi_{0}\left(t\right)}{2\pi {\left(t-u_{k}\right)}^{3}}}\text{exp}\left\{-\frac{1}{2}\frac{\xi_{0}\left(t\right){\left[t-u_{k}-\mu_{\text{HP}}\left(t,{ℋ}_{t},\xi \left(t\right)\right)\right]}^{2}}{\mu_{HP}{\left(t,{ℋ}_{t},\xi \left(t\right)\right)}^{2}\left(t-u_{k}\right)}\right\}$$ (1)

where ${ℋ}_{t}=(u_{k},HP_{k},{\text{HP}}_{k-1},...,{\text{HP}}_{k-K+1})$, $\xi (t)=\{g_{0}(t),g_{1}(t),$$\xi_{0}(t)\}$ is the time-resolved parameter vector, and $\xi_{0}(t)$>0 is the shape parameter of the inverse-Gaussian distribution.

Through the following j^th^-order discrete-time orthonormal Laguerre function:

$$\phi_{j}\left(n\right)=a^{\frac{n-j}{2}}{\left(1-a\right)}^{\frac{1}{2}}\sum_{i=0}^{\text{j}}{\left(-1\right)}^{i}\left(\begin{array}{c} n\\ i \end{array}\right)\left(\begin{array}{c} j\\ i \end{array}\right)\alpha^{j-1}{\left(1-\alpha \right)}^{i}$$

It is possible to calculate the Laguerre filter output $l_{j}\left(t\right)$ as follows:

$$l_{j}\left(t\right)=\sum_{n=1}^{\tilde{N}\left(t\right)}\phi_{j}\left(n\right)HP_{\tilde{N}\left(t\right)-\text{n}}$$

where $j=\tilde{N}\left(t\right)$ is the index of the previous heartbeat event before time t, $N\left(t\right)=\text{max}\left\{k:u_{k}\leq t\right\}$ is the sample path of the counting process of the HP interval series, $\tilde{N}(t)=N(t^{-})={\text{lim}}_{\tau \to \;t^{-}}N(\tau )=\text{max}\{k:u_{k}<t\}$, and:

$$\begin{array}{l} \mu_{\text{HP}}(t,{ℋ}_{t},\xi (t))=g_{0}\left(t\right)+\sum_{j=o}^{{\text{P}}_{\text{symp}}}g_{1}\left(j,t\right)l_{j}\left(t\right)\\ \;\;\;\;\;\;\;\;\;\;\;\;\;\;\;\;\;\;\;\;\;\;\;\;\;\;\;\;\;\;\;\;\;\;\;\;\;\;\;\;+\sum_{j=P_{symp+1}}^{P_{parasymp}}g_{1}\left(j,t\right)l_{j}\left(t\right) \end{array}$$

SAI and PAI estimations are based on the orthonormal Laguerre expansion of the autoregressive components (Valenza et al., 2018a, 2018b). Considering ${\text{P}}_{\text{symp}}=2$ and $P_{parasymp}=9$, SAI is computed as:

$$SAI(t,\xi (t))=\psi_{So}+\sum_{j=1}^{{\text{P}}_{\text{symp}}}\psi_{S_{j}}g_{1}(j,t)$$ (2)

Similarly, PAI is computed as

$$PAI(t,\xi (t))=\psi_{Po}+\sum_{j=1}^{P_{parasymp}}\psi_{P_{j}}g_{1}(j+{\text{P}}_{\text{symp}},t)$$ (3)

The coefficients ψ are used in accordance with our earlier findings (Valenza et al., 2018b), where a final normalization involves dividing SAI(t,ξ(t)) and PAI(t,ξ(t)) by the square of $\mu_{\text{HP}}(t,{ℋ}_{t},\xi (t))$ (Valenza et al., 2018a).

We calculate the time-varying parameter vector ξ(t) that enhances the local log-likelihood using the Newton–Raphson method with the local observation of HP intervals of period W=70 s. The model is repeatedly revised every 5 ms.

The time-resolved computation of SAI and PAI series from heartbeat data is publicly available at http://www.saipai-hrv.com/.

**Fig. 1.**
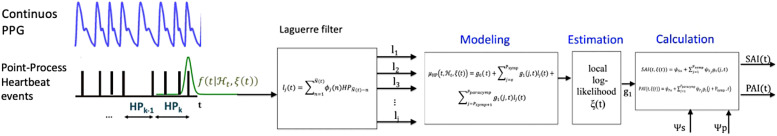
Integrated analysis workflow for SAI and PAI estimation.

A block scheme showing the overall SAI and PAI estimation pipeline is illustrated in the Figure 1 below

### 2.5 Joint SAI/PAI and rs-fMRI analysis

**Fig. 2.**
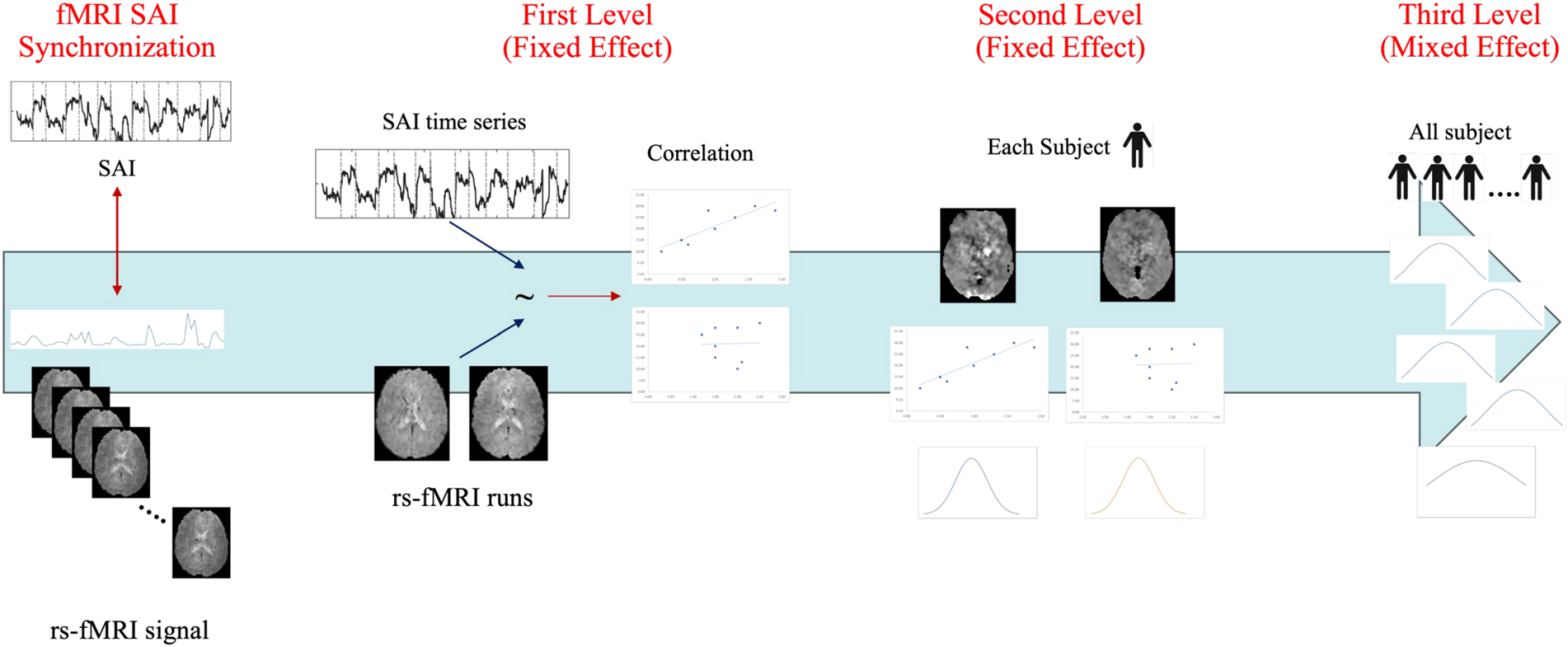
Integrated analysis workflow for joint SAI and PAI signals with rs-fMRI signals. The joint SAI-fMRI analysis is specifically illustrated. The joint SAI-fMRI analysis is similarly performed by substituting SAI with PAI series.

An overview of the joint SAI/PAI and rs-fMRI analysis is provided in Figure 2. To perform the joint analysis, the SAI and PAI time series were first resampled at the timing of the fMRI time series by synchronizing their values with each fMRI timepoint (i.e., with temporal spacing equal to the repetition time TR). Synchronized SAI and PAI series were then convolved with a double gamma hemodynamic response (Lindquist et al., 2009) for further processing. The joint analysis has three levels.

- Level 1. In a first-level, fixed-effects analysis, for each regressor (SAI and PAI), we generated both positive and negative contrast maps;
- Level 2. A second-level fixed-effects analysis was employed for each regressor to summarize within-subject effects across the two sessions conducted on the same day;
- Level 3. The individual parameter estimates along with their variances were passed up to a third (group) level via a mixed-effects analysis (FLAME, FEAT, FSL).

We performed a voxel-wise analysis, followed by a cluster-based correction analysis covering the entire brain. Successively, we utilized the “atlasquery” function from the FSL library to link a statistically significant voxel to a specific region. This automatic localization procedure was further enhanced by visually inspecting the resulting maps. The Harvard-Oxford cortical and subcortical atlas were used as input for the “atlasquery” tool.

We processed the Gaussianized T/F statistic maps by applying a threshold of 2.3. We then performed a whole-brain cluster-based correction analysis, maintaining a significance level at alpha=0.05, as suggested by Worsley (2012).

For all analyses, we employed the fMRI Expert Analysis Tool (FEAT v.5.90, FSL), as well as the brain mask provided in the FSL library (2 mm isotropic, MNI space).

## 3 Results

In this study, we aimed at broadening and refining our knowledge of the sympathetic Central Autonomic Network (CAN). To achieve this, we used the resting-state functional Magnetic Resonance Imaging (rs-fMRI) data of 34 healthy volunteers from the Human Connectome Project (HCP), along with the recently established Sympathetic Activity Index (SAI). We also evaluated the parasympathetic CAN using the Parasympathetic Activity Index (PAI). After calculating the SAI and PAI indices for each participant, we analyzed the correlation, both positive and negative, between the Blood Oxygen Level-Dependent (BOLD) rs-fMRI signal and the two indices. The results of this analysis are detailed in the subsequent paragraph.

### 3.1 Sympathetic CAN: SAI-fMRI analysis

**Fig. 3.**
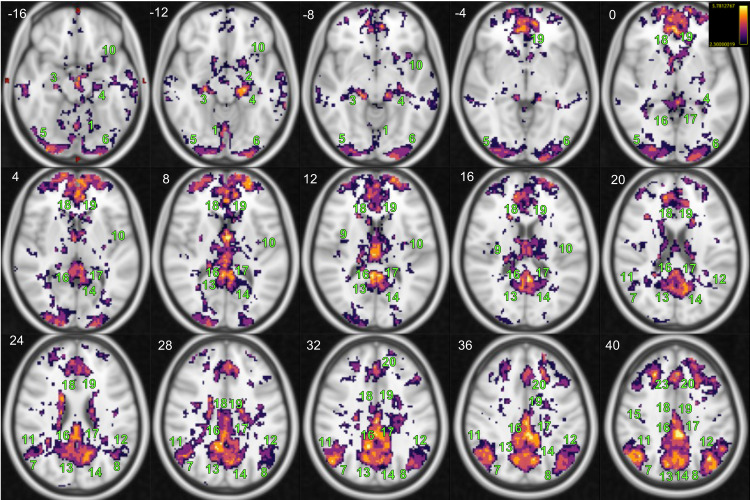
Brain regions demonstrating significant positive correlations with the estimated instantaneous parasympathetic autonomic outflow via SAI are displayed in whole-brain corrected Z maps (Z > 2.3) superimposed on the standard 2 mm MNI T1-weighted image (in Axial plane). No significant results were found in the opposite contrast. The z coordinate (cm) is in the superior left corner of each slice. Number legends: 1) Vermis 6; 2) Left Amygdala; 3) Right Hippocampus; 4) Left Hippocampus; 5) R-Lateral Occipital Cortex inferior division; 6) L-Lateral Occipital Cortex inferior division; 7) R-Lateral Occipital Cortex superior division; 8) L-Lateral Occipital Cortex superior division; 9) R-Insular Cortex; 10) L-Insular Cortex; 11) R-Angular Gyrus; 12) L-Angular Gyrus; 13) R-Precuneus Cortex; 14) L-Precuneus Cortex; 15) R-Precentral Gyrus; 16) R-Posterior Cingulate Gyrus; 17) L-Posterior Cingulate Gyrus; 18) R-Anterior Cingulate Gyrus; 19) L-Anterior Cingulate Gyrus; 20) L-Superior Frontal Gyrus; and 23) R-Superior Frontal Gyrus.

In a group-level analysis, we found a statistically significant positive association between SAI and rs-fMRI signals in several brain regions (Fig. 3), while no negative associations were detected in the opposite contrast (Fig. 3). These associations are widespread in the cortical, subcortical, and cerebellar cortices (for complete results, see Supplementary Tables 1 and 2). In particular, we found a positive SAI-rsfMRI association in numerous regions highlighted in previous studies (Beissner et al., 2013; Benarroch, 2020; Gertler et al., 2020; Macefield & Henderson, 2019; Nakamura & Morrison, 2022), such as the bilateral insular cortex, bilateral PFC, cingulate gyrus (anterior, mid and posterior), frontal pole, supplementary motor, and superior frontal gyrus. In the parietal lobe, we found a positive bilateral association in the postcentral gyrus, precuneus, supramarginal gyrus, and left superior parietal lobule. Interestingly, in the temporal lobe and in the occipital lobe, we found a positive association bilaterally in the parahippocampal gyrus, fusiform cortex, lateral occipital cortex, lingual gyrus, and calcarine gyrus, as well as in most of the temporal gyri and occipital cortex. Similarly, in subcortical structures, we found a positive association between the rs-fMRI signal and the SAI bilaterally in the thalamus, hippocampus, basal ganglia, cerebellar cortex, and brainstem. a positive association between the rs-fMRI signal and the SAI bilaterally in the thalamus, hippocampus, basal ganglia, cerebellar cortex, and brainstem.

### 3.2 Parasympathetic CAN: PAI–fMRI analysis

**Fig. 4.**
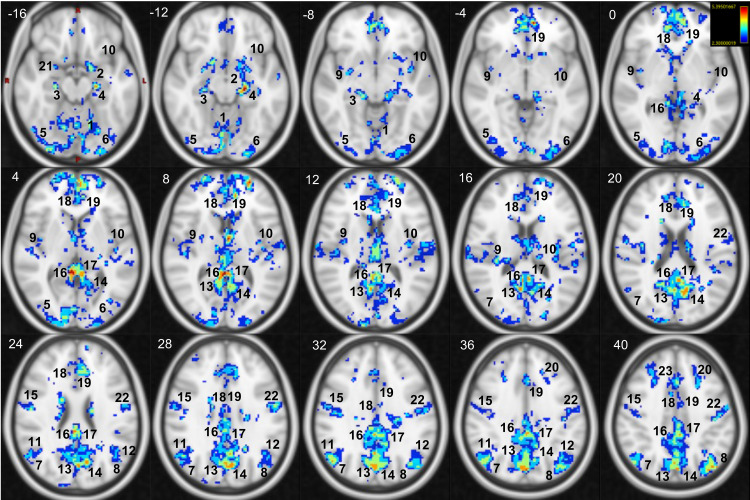
Brain regions exhibiting negative correlations with instantaneous parasympathetic autonomic outflow as estimated through PAI are depicted in the thresholded Z maps (Z>2.3) superimposed on the standard 2 mm MNI T1-weighted image (in Axial plane). No significant results were observed for the opposite contrast. The z coordinate (cm) is in the superior left corner of each slice. Number legends: 1) Vermis 6; 2) Left Amygdala; 3) Right Hippocampus; 4) Left Hippocampus; 5) R-Lateral Occipital Cortex inferior division; 6) L-Lateral Occipital Cortex inferior division; 7) R-Lateral Occipital Cortex superior division; 8) L-Lateral Occipital Cortex superior division; 9) R-Insular Cortex; 10) L-Insular Cortex; 11) R-Angular Gyrus; 12) L-Angular Gyrus; 13) R-Precuneus Cortex; 14) L-Precuneus Cortex; 15) R-Precentral Gyrus; 16) R-Posterior Cingulate Gyrus; 17) L-Posterior Cingulate Gyrus; 18) R-Anterior Cingulate Gyrus; 19) L-Anterior Cingulate Gyrus; 20) L-Superior Frontal Gyrus; 21) Right Amygdala; 22) L-Precentral Gyrus; and 23) R-Superior Frontal Gyrus.

Group-level analysis reveals a statistically significant negative association between PAI and rs-fMRI activity in several regions belonging to both the cortex and subcortical structures (Fig. 4, complete results in Supplementary Tables 3 and 4). No positive associations were observed in the opposite contrast. These areas include the bilateral insula, PFC, cingulate gyrus (anterior, mid and posterior), superior and inferior frontal gyrus, and supplementary motor area. Moreover, negative associations were found in the parietal lobe, the bilateral postcentral gyrus, precuneus, supramarginal gyrus, angular gyrus, and superior parietal lobule. In addition, similar results were observed in the temporal and occipital lobes: bilaterally in the superior and middle temporal gyrus, in the parahippocampal gyrus (posterior division), in the fusiform cortex, and in the primary and secondary visual cortex. Analogous results were also found in subcortical regions: bilaterally in the thalamus, hippocampus, basal ganglia, cerebellar cortex, and brain stem.

### 3.3 Common regions in the parasympathetic/sympathetic CAN

**Fig. 5.**
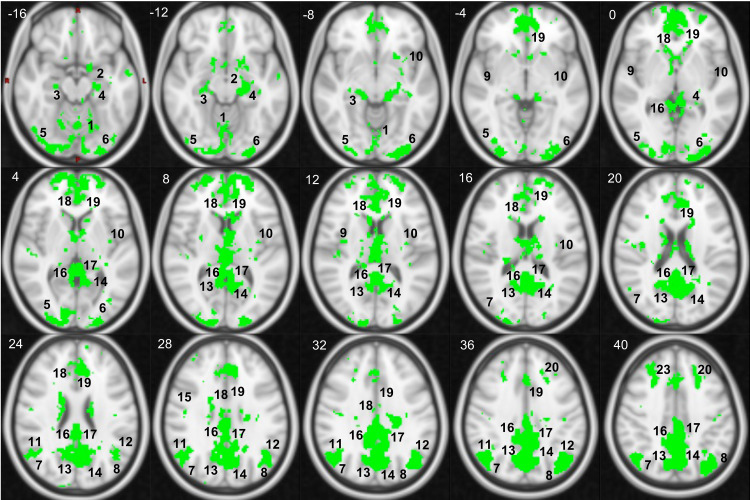
Brain regions demonstrating significant correlations between resting state activity and autonomic nervous system activity, as measured by SAI (Sympathetic Autonomic Index) and PAI (Parasympathetic Autonomic Index), shown as a mask superimposed on a standard 2 mm MNI T1-weighted image (in Axial plane). The z coordinate (cm) is in the superior left corner of each slice. Number legends: 1) Vermis 6; 2) Left Amygdala; 3) Right Hippocampus; 4) Left Hippocampus; 5) R-Lateral Occipital Cortex inferior division; 6) L-Lateral Occipital Cortex inferior division; 7) R-Lateral Occipital Cortex superior division; 8) L-Lateral Occipital Cortex superior division; 9) R-Insular Cortex; 10) L-Insular Cortex; 11) R-Angular Gyrus; 12) L-Angular Gyrus; 13) R-Precuneus Cortex; 14) L-Precuneus Cortex; 15) R-Precentral Gyrus; 16) R-Posterior Cingulate Gyrus; 17) L-Posterior Cingulate Gyrus; 18) R-Anterior Cingulate Gyrus; 19) L-Anterior Cingulate Gyrus; 20) L-Superior Frontal Gyrus; 21) Right Amygdala; 22) L-Precentral Gyrus; and 23) R-Superior Frontal Gyrus.

Figure 5 highlights the regions that showed both a significant positive association between SAI rs-fMRI and significant negative associations between PAI and rs-fMRI. A detailed list of brain locations included in these regions is available in the Supplementary Materials (Supplementary Table 5, cortical, and Supplementary Table 6, subcortical and cerebellum). Nonoverlapping regions are also reported in the Supplementary Material, Tables 7 and 8. Overall, most SAI-related brain areas coincided with PAI-related brain areas, albeit with opposite signs in the correlations between dynamic autonomic indices and rs-fMRI dynamics. In terms of nonoverlapping regions, we found that the posterior division of the right supramarginal gyrus, the posterior division of the middle temporal gyrus, the posterior division of the inferior temporal gyrus (bilaterally), the anterior division of the right parahippocampal gyrus, and the bilateral pallidum showed only a significant positive association between the SAI and rs-fMRI BOLD signal. Instead, the right superior parietal lobule and (bilaterally) anterior division of the supramarginal gyrus showed only a significant positive association between the PAI and rs-fMRI BOLD signal.

## 4 Discussion

By using heartbeat dynamics and the recently defined SAI (Valenza et al., 2018a, 2018b) to noninvasively proxy instantaneous sympathetic activity, we uncovered the sympathetic-related parts of the CAN through high-resolution rs-fMRI data from the HCP database. Complementarily, we exploit PAI estimates to investigate functional correlates of parasympathetic activity. We found a widespread correlation between the BOLD signal and the two indices (SAI: positive association, PAI; negative association) in both cortical (in all lobes) and subcortical areas, including the cerebellum and brainstem. The associations between BOLD activity and SAI or PAI were seen to be in opposite directions, which may reflect the reciprocal, complementary activity of the sympathetic and parasympathetic nervous systems. In the following text, we discuss brain regions we found in the sympathetic CAN (according to SAI) and parasympathetic CAN (according to PAI). It is important to highlight that the parasympathetic CAN, determined through correlation analysis between fMRI and the PAI series, can be compared with the CAN that was earlier identified through HRV analysis, grounded on the spectral parameter (High Frequency power) (Chang et al., 2013; Nikolaou et al., 2016; Sclocco, Kim, et al., 2016). However, it is noteworthy that the sympathetic CAN has not been singularly identified using non-invasive data in previous studies. The innovative aspect of our research precisely resides in the detection of the sympathetic CAN during the resting-state conditions, achieved by employing non-invasive measures (HRV series from Photoplethysmography signals). We are confident that this discovery significantly enriches existing knowledge and underscores the distinct contribution of our study to the nascent field of brain-heart and brain-body investigation.

We opted to use the preprocessed data provided by the HCP consortium. These data have undergone comprehensive denoising through the ICA-FIX strategy, specifically tailored for HCP data (Salimi-Khorshidi et al., 2014). The strategy aims at eliminating various noise sources, including physiological noise. Although the exact extent to which this method eradicates physiological variance in the BOLD signal remains unclear, there is a possibility of some residual contamination in our findings. Nonetheless, considering that our investigation revolves around the effect of interest regressors derived from the plethysmography signal, we concluded it was best not to depict physiological noise by using no interest regressors (for instance, RETROICOR, RVCOR (Chang and Glover, 2009)) obtained from the same signal. This led us to adopt an impartial and evidence-based methodology. Our approach, which was not guided by specific a priori hypotheses or predefined areas of interest, could however potentially pose a limitation in terms of specificity. Specifically, it might hinder the clarification of the exact functions of the identified brain structures in relation to the CAN. Future studies, possibly utilizing seed-based or task-driven data, will be required to comprehensively address these aspects.

The amygdala has been extensively researched for its role in emotional processing, particularly in the evaluation of negative stimuli (Davern & Head, 2011; Nestler et al., 2002), which is known to be closely linked to sympathetic control. In recent years, studies have explored the amygdala’s participation in regulating cognitive processes such as attention, perception, and memory (LeDoux, 2007). For example, neuroimaging studies have shown that the consolidation of emotionally charged memories into long-term memory is facilitated by the integration of neuromodulatory processes in the basolateral amygdala, which then projects to brain regions responsible for memory consolidation (McGaugh, 2004). Furthermore, recent research has indicated the amygdala’s involvement in parasympathetic control, potentially linked to the requirement of balancing increased sympathetic and decreased parasympathetic output in response to adverse stimuli.

The insula is a complex structure with multiple subregions that have connections to the cingulate cortex (Deen et al., 2011). Its role in autonomic regulation and interoceptive feedback has been extensively studied (Craig, 2002; Critchley et al., 2000). Our findings, showing a negative relationship between the insula’s rs-fMRI BOLD signal and PAI, align with previous research, indicating that a heightened autonomic response is associated with a decreased insula response during motion sickness induced by visual stimuli (Sclocco, Beissner, et al., 2016). Another study utilizing ASL to measure regional cerebral blood flow during rest found a negative correlation between resting HF-HRV and perfusion in the frontal operculum (Allen et al., 2015). These findings suggest that the insula plays a role in suppressing the premotor brain stem nuclei that control cardiovagal autonomic output (Sclocco, Kim, et al., 2016). The insula integrates nociceptive and viscerosensory signals, as well as modulates sympathetic and parasympathetic activity through pathways that are regulated by the hypothalamus and brainstem (Benarroch, 2012; Critchley, 2005; Oppenheimer & Cechetto, 2016). Aside its well-established role in parasympathetic activity (Beissner et al., 2013; Quadt et al., 2022), recent studies found that the insula’s activity is closely linked to muscle sympathetic nerve activity (MSNA) during rest. (Fatouleh et al., 2014; Kobuch et al., 2018; Lundblad et al., 2014; Macefield & Henderson, 2019). Macefield et al. argued that this region can regulate sympathetic outflow through afferent projection of the baroceptor from the NTS to the posterior insula, which, in turn, projects to the anterior insula (Macefield & Henderson, 2023). Moreover, the latter sends projections also to numerous hypothalamic nuclei (ventromedial and dorsomedial hypothalamus respectively), which then sends projections to different columns of the midbrain PAG (Macefield & Henderson, 2019, 2023).

The central role of the hypothalamus in the CAN has been widely described (Benarroch, 2012, 2020; Macefield & Henderson, 2019). The hypothalamus contributes to cardiovascular control through its connection to the PAG, and a recent connectivity analysis (James et al., 2013) showed that the BOLD signal of the ventromedial hypothalamus (VMH) covaried with both cortical (dorsolateral PFC, precuneus, and insula) and brainstem regions (bilateral RVLM). Therefore, as Macefield and Henderson pointed out (Macefield & Henderson, 2019), the network composed by insula, hypothalamus, PAG, and RVLM has an important role in controlling the sympathetic outflow in humans at rest.

Along with the insula, the BOLD signal of other cortical regions such as dorsolateral PFC, PCC, and precuneus exhibited correlation with MSNA burst (James et al., 2013; Macefield & Henderson, 2016). These regions have also shown functional coupling with either the hypothalamus or the RVLM and the PCC sends efferent projections to the PAG (James et al., 2013; Macefield & Henderson, 2016, 2019, 2020). The PCC and the precuneus are part of the DMN (Duncan, 2010). Macefield and Henderson (Macefield & Henderson, 2019) suggested that these regions, and particularly the precuneus, could influence sympathetic outflow that the RLVM exert during awake period using an indirect pathway (through either the PAG, the VMH, or the DMH) (Macefield & Henderson, 2019, 2023).

In addition to the PCC, other areas of the cingulate cortex influence autonomic activity (Benarroch, 2012, 2020). Indeed, the cingulate cortex plays a significant role in a range of brain processes, including emotions, memory, and actions (Rolls, 2019). The cingulate gyrus and pregenual anterior cingulate are particularly relevant to the regulation of pain and emotional processing (Critchley et al., 2003; Hagemann et al., 2003). Research has suggested that the mid-cingulate plays a crucial role in the regulation of autonomic activity (Luu & Posner, 2003; Medford & Critchley, 2010), while the anterior part is involved in pain processing (Farrell et al., 2005) and fear perception (Mechias et al., 2010). Two studies by Critchley et al. (Critchley, 2005; Critchley et al., 2003) found that the low-frequency component of HRV and the BOLD signal in the anterior and mid-cingulate gyrus are correlated when performing mental arithmetic and isometric exercise. Additionally, the BOLD signal was able to predict changes in HR while processing facial expressions. The cingulate cortex has high somatosensory capabilities (Vogt, 2005) and its responses involve thalamic neurons that obtain information from the parabrachial nuclei, which are a key gateway for vagal activity (Cechetto, 2014). Additionally, slow vascular and neural BOLD oscillations (~0.1 Hz) originating in the cingulum contribute to heart rate variability (Pfurtscheller et al., 2017, 2021). It is worth mentioning that stimulation of the vagal nerve reduces activity in the cingulate gyrus (Henry, 2002; Nahas et al., 2007).

Hippocampal activity has been linked to autonomic functioning (Henderson et al., 2002; Macefield et al., 2006), emotional processing, and other psychiatric dysfunctions (Critchley et al., 2000; Williams et al., 2004). Particularly, BOLD signal intensity reduction was seen in the hippocampus, correlating with reduced activity in medullary structures (Macefield et al., 2006). In this paper, Macefield et al. argued that the signal modification in the hippocampus could be related to the generation or regulation of sympathetic outflow, since the hippocampus has an indirect pathway to the adrenal gland and stellate ganglion that are involved in sympathetic regulation (Westerhaus & Loewy, 2001).

The temporal gyrus is involved in emotional and social processing (Basile et al., 2013; Lane, 2008; Nicotra et al., 2006; O’Connor et al., 2007) and exhibits inverse correlation with vagal activity during grip tasks (Napadow et al., 2008). Furthermore, the temporal gyrus exhibits nonlinear dynamics (Valenza, Passamonti, et al., 2020; Valenza et al., 2017) in individuals with high levels of anxiety, as indicated by fMRI analysis (Tolkunov et al., 2010) and is activated after stimulation of the vagal nerve (Nahas et al., 2007). Additionally, Wei et al. (Wei et al., 2018) found that HF-HRV negatively correlated with gray matter in the superior temporal gyrus and parahippocampal gyrus in healthy individuals.

The activity of the frontal orbital cortex has been found to negatively correlate with changes in heart rate variability, suggesting its involvement in fear regulation mechanisms (Makovac et al., 2015) and autonomic dysregulation in individuals with generalized anxiety disorder (Makovac et al., 2016). Moreover, frontal pole activity has also been linked to autonomic control mechanisms (Y. Nagai et al., 2004) and retrospective memory (Okuda et al., 2003). The uperior and middle frontal gyri have been linked to cardiac vagal regulation, which is related to various actions, such as volitional and affective actions (Critchley et al., 2000; Kimmerly et al., 2005; Matthews et al., 2004).

The activity of the angular gyrus has been observed to be altered by brief periods of meditation, which serves as a hallmark demonstration of the relationship between the brain and heart (Tang et al., 2009). The lateral occipital cortex is implicated in the autonomic responses elicited by acupuncture (Beissner et al., 2012) and the expression of emotions (Nicotra et al., 2006).

Surprisingly, we did not find a positive correlation between SAI and the BOLD signal in the rostral ventrolateral medulla (Cersosimo & Benarroch, 2013; Kumagai et al., 2011; Macefield & Henderson, 2019, 2020) and PAG. (Benarroch, 2012, 2020; Quadt et al., 2022). One possible explanation for our null findings could be related to the difficulties in capturing these structures while using fMRI due to limits related to image resolution (Linnman et al., 2012) or differences in experimental paradigms as opposed to resting state.

Our results are slightly at odds with previous accounts on the CAN (Beissner et al., 2013; Benarroch, 2012, 2020). Indeed, we found widespread associations between the BOLD signal and both SAI (positive) and PAI (negative) in several regions that are also part of the DMN (e.g., medial preforntal cortex, anterior cingulate gyrus, angiular gyrus, etc.), which are commonly thought to be mostly related to parasympathetic activity. Similarly, we found fMRI associations with both SAI and PAI in regions that also belong to the salience and executive networks (e.g., dorsolateral PFC, posterior parietal cortex), which are commonly described as mostly associated with sympathetic activity. On the one hand, the set of regions that our study outlines as bars for the CAN expands well beyond the regions canonically thought to belong to this complex network (Benarroch, 2012, 2020), in accordance with our previous findings about the association between dynamic HF-HRV estimates and BOLD activity (Valenza et al., 2019). On the other hand, the neural correlates of the nonspecific skin conductance response, a measurement thought to be linked to sympathetic activity, seem to be mostly localized at a cortical level (including the parietal lobe and cingulate cortex), with no involvement of the cerebellum, thalamus, amygdala, or brainstem at rest (Gertler et al., 2020).

There are several, non-mutually exclusive explanations for interpreting our results. First, our study was not limited to a priori region of interest (ROI) analysis or to an *a priori* sub-selection of brain regions. Second, most previous studies that measure parasympathetic and especially sympathetic activity in a noninvasive manner suffer from a number of limitations (Valenza et al., 2018b), which have been largely overcome by the use of SAI and PAI estimates (Valenza, Faita, et al., 2020). We, therefore, hypothesize that the true extension of the CAN network related to parasympathetic/sympathetic activity could have been previously underestimated. For instance, a few recent fMRI studies focused on visual brain regions (Chang et al., 2013; Nikolaou et al., 2016; Sclocco, Kim, et al., 2016) that employed parasympathetic and sympathovagal indicesand found similar results to ours. Nikolaou et al. (2016) found significant correlations between HR and dynamic connectivity values within the visual and somatosensory resting-state networks, and Chang et al. (2013) showed that low-frequency HRV correlated with functional connectivity between dorsal ACC and the amygdala (used as seed regions) and the parieto-occipital cortex.

Interestingly, we also found associations between both SAI and PAI and the BOLD signal bilaterally in the cerebellar cortex. The influence of cerebellar structures during postural changes on the cardiovascular response has been highlighted in conscious rabbits (Nisimaru et al., 1998). Moreover, in unconscious cats (Barman & Gebber, 2009), causal alteration of lobule IX influenced naturally occurring cardiac sympathetic nerve discharges. In humans, the cerebellum, apart from hosting well-known CAN regions (Benarroch, 2012, 2020), appears to be involved in volitional and nonvolitional baroreflex control of autonomic cardiovascular function (Kimmerly, 2017). Additionally, the cerebellum, along with the angular gyrus and the precunes, has previously been reported in a recent neuroimaging meta-analysis that studied CAN regions (Beissner et al., 2013) and in a study from Macefield et al. (Macefield et al., 2006), where they found correlation between BOLD signal in the cerebellum and MSNA. Although its role in CAN functioning is still not completely elucidated, Macefield et al speculated that its role in autonomic function could be related to its role in motor control (Macefield et al., 2006).

A recent study investigated the effect of regular endurance training on six core regions of the CAN (ventromedial PFC, dorsolateral ACC, left/right amygdala, and left/right insula) comparing resting state functional connectivity (rs-FC) between a group of 20 endurance athlete and 21 non-athletes (de la Cruz et al., 2022). Interestingly, when they used the dorsolateral ACC as seed, they found significant differences between the group in the rs-FC in a large cluster at the level of the primary sensorimotor cortex (S1/M1). Moreover, differences in the rs-FC of sensorimotor regions were highlighted also when using the insula as seed.

Finally, we note that differences in findings related to sympathetic and parasympathetic CANs, as uncovered through different autonomic proxies, may be linked to the existence of several autonomic controls organized into parallel neural circuits (Veerakumar et al., 2022).

While one would expect more activations in the brainstem, this is more likely due to the limited signal-to-noise ratio in brainstem imaging (Sclocco et al. 2019) as well as coregistration difficulties in this area. As these factors are potentially limiting the exploration of their functional implications within the identified sympathetic and parasympathetic CANs, future research will focus on an in-depth brainstem analysis to uncover the brainstem’s role in sustaining CANs as well as the overall brain connectivity.

## 5 Conclusion

Through the concurrent analysis of rs-fMRI data and recently designed, accurate estimators of dynamic ANS activity separated into sympathetic and parasympathetic contributions, this study highlights active and distributed regulation by central autonomic networks of both parasympathetic and, importantly, sympathetic activity at rest. These findings extend well beyond brain areas previously hypothesized to be involved in such processes, such as important nodes of the DMN and executive network, and include possible overlaps between the brain regions involved in driving and integrating the two systems, for example, the amygdala, insular cortex, and cingulate gyrus. Further research is necessary, either using drugs, tasks, or brain stimulation, to determine the impact of a modulation of either the sympathetic or parasympathetic system on the CAN’s response related to cardiovascular control.

## Supplementary Material

Supplementary Material

## Data Availability

The Human Connectome Data used for this study are publicly available at: http://www.humanconnectomeproject.org
