# Supplementary material for: Sympathetic and parasympathetic central autonomic networks
Source: Imaging Neurosci (Camb). 2024 Feb 28;2:imag_2_00094. doi: 10.1162/imag_a_00094 (PMC12224469; doi:10.1162/imag_a_00094)
Supplement: Supplementary Material [file imag_a_00094-supp.pdf]

Sympathetic and Parasympathetic Central-Autonomic Networks

Supplementary Materials

A. Subject selection

The final list of the 34 subjects selected from the HCP U100 dataset and used in this study (from each subject, we employed exactly 2 sane-day resting-state fMRI runs with opposite phase encoding direction) is as follows:

100307, 103111, 103818, 105014, 108828, 111716, 118730, 118932, 120111, 122620, 128127, 128632, 129028, 131217, 131722, 133019, 135225, 136833, 148335, 148840, 149741, 151526, 151627, 154734, 156637, 178950, 188347, 189450, 199655, 211720, 756055, 792564, 856766, 899885.

Subjects were excluded because of low-quality / missing cardiac signals (in at least one of the 2 resting-state runs collected on the same day):

**Reason from exclusion**	**Subject IDs**
Incomplete signal	101309, 118528, 118528, 140925
Noisy signal[1]	101915, 106016, 110411, 111312, 113619, 113922, 114419, 122317, 124422, 125525, 127630, 130316, 144832, 147737, 149337, 153025, 159340, 161731, 162733, 163129, 214423, 221319, 280739, 298051, 499566, 672756
Lack of Heartbeat Interval variability[2]	103414, 105015, 115320, 116524, 117122, 123925, 130013, 133928, 135932, 139637, 146432, 149539, 176542, 192540, 201111, 212318, 366446, 397760, 414229, 654754, 857263
Other	101107, 123117

[1]"Noisy signals": signal that contained multiple artifacts such that beats could not be accurately recognized and the point process model did not converge.

[2]"Lack of Heartbeat Interval variability": subjects whose heartbeat interval series showed a variability extremely close to zero (possibly due to MR acquisition artifacts and/or problems in data acquisition/storage, which interfered with reliable peak detection).

Group level analysis results tables

Brain Regions	Z score	X*	Y*	Z*
Frontal Lobe				
L-Frontal Pole	5.709	57	91	39
R-Frontal Pole	5.007	38	96	37
L-Superior Frontal Gyrus	5.721	56	77	62
R-Superior Frontal Gyrus	5.708	33	78	57
L-Middle Frontal Gyrus	4.965	64	68	64
R-Middle Frontal Gyrus	5.731	33	78	56
L-Frontal Orbital Cortex	3.331	60	68	27
R-Frontal Orbital Cortex	3.401	38	68	29
L-Frontal Medial Cortex	4.949	48	87	31
R-Frontal Medial Cortex	4.180	39	88	32
L-Paracingulate Gyrus	4.941	49	85	41
R-Paracingulate Gyrus	5.667	43	87	35
L-Cingulate Gyrus, anterior division	5.705	48	84	37
R-Cingulate Gyrus, anterior division	4.894	44	84	35
L-Cingulate Gyrus, posterior division	5.731	50	43	55
R-Cingulate Gyrus, posterior division	5.735	42	39	50
L-Juxtapositional Lobule Cortex	3.412	49	57	62
R-Juxtapositional Lobule Cortex	3.419	42	56	67
L-Precentral Gyrus	4.877	46	52	72
R-Precentral Gyrus	3.432	28	53	70
Parietal Lobe				
L-Postcentral Gyrus	4.152	48	45	74
R-Postcentral Gyrus	3.446	30	48	69
L-Superior Parietal Lobule	3.392	60	34	57
L-Supramarginal Gyrus posterior division	4.916	70	38	63
R-Supramarginal Gyrus posterior division	3.415	21	40	60
L-Angular Gyrus	4.996	70	36	57
R-Angular Gyrus	5.002	22	36	54
L-Precuneous Cortex	5.759	48	29	57
R-Precuneous Cortex	5.717	40	36	51
L-Central Opercular Cortex	2.649	66	55	47

R-Central Opercular Cortex	3.412	26	57	47
L-Parietal Operculum Cortex	2.664	65	49	48
R-Parietal Operculum Cortex	2.606	25	53	45
Temporal Lobe				
L-Superior Temporal Gyrus anterior division	2.524	76	61	32
L-Superior Temporal Gyrus posterior division	2.587	74	47	35
R-Superior Temporal Gyrus posterior division	2.551	14	59	31
L-Middle Temporal Gyrus anterior division	3.435	74	59	27
R-Middle Temporal Gyrus anterior division	3.385	18	62	25
L-Middle Temporal Gyrus posterior division	4.189	77	54	27
R-Middle Temporal Gyrus posterior division	4.186	15	53	28
L-Middle Temporal Gyrus temporooccipital part	3.365	78	41	31
R-Middle Temporal Gyrus temporooccipital part	2.682	16	43	34
L-Inferior Temporal Gyrus posterior division	3.396	76	44	26
R-Inferior Temporal Gyrus posterior division	4.149	15	48	27
R-Inferior Temporal Gyrus temporooccipital	3.382	16	38	25
L-Inferior Temporal Gyrus temporooccipital	3.373	74	39	27
L-Parahippocampal Gyrus anterior division	3.398	55	52	25
R-Parahippocampal Gyrus anterior division	2.583	35	54	24
L-Parahippocampal Gyrus posterior division	4.876	53	48	30
R-Parahippocampal Gyrus posterior division	4.826	36	49	31
L-Temporal Fusiform Cortex, posterior division	2.538	58	44	24
L-Temporal Occipital Fusiform Cortex	2.548	57	31	28
R-Temporal Occipital Fusiform Cortex	2.548	31	33	27
L-Heschls Gyrus	2.612	63	50	41
R-Heschls Gyrus	3.335	24	53	42
L-Planum Temporale	2.631	66	43	43
R-Planum Temporale	2.666	18	53	38
L-Planum Polare	2.648	66	59	28
R-Temporal Fusiform Cortex, posterior division	2.509	32	48	23
L-Subcallosal Cortex	3.393	47	71	34
R-Subcallosal Cortex	3.340	44	70	35
Occipital Lobe				
L-Occipital Fusiform Gyrus	4.145	56	18	26

R-Occipital Fusiform Gyrus	4.191	30	19	28
L-Lateral Occipital Cortex superior division	5.776	63	30	57
R-Lateral Occipital Cortex superior division	5.781	24	32	56
L-Lateral Occipital Cortex inferior division	4.186	61	18	34
R-Lateral Occipital Cortex inferior division	4.956	29	18	28
L-Intracalcarine Cortex	2.601	50	20	36
R-Intracalcarine Cortex	2.651	37	22	42
L-Cuneal Cortex	4.051	45	24	53
R-Cuneal Cortex	4.095	43	25	54
L-Lingual Gyrus	3.386	46	20	31
R-Lingual Gyrus	4.185	42	18	31
L-Supracalcarine Cortex	3.338	49	30	44
R-Supracalcarine Cortex	2.510	44	20	38
R-Occipital Pole	4.237	35	16	39
L-Occipital Pole	4.960	54	12	35
Insula				
L-Insular Cortex	3.368	62	68	31
R-Insular Cortex	2.610	28	60	42

Table 1. From left to right: brain regions (from the Harvard-Cortical atlas) that shows positive correlations between the resting state BOLD signal and the SAI; Z-score; X, Y, Z voxel coordinates in the standard MNI 2 mm space

**Brain Regions**	**Z-score**	**X***	**Y***	**Z***
Left Thalamus	5.693	46	57	41
Right Thalamus	5.603	43	52	42
Left Caudate	4.157	51	72	37
Right Caudate	4.964	36	66	47
Left Putamen	4.158	55	67	30
Right Putamen	3.424	30	60	40
Left Pallidum	2.664	54	58	36
Right Pallidum	2.654	36	61	38
Left Hippocampus	4.844	55	50	30
Right Hippocampus	4.851	33	51	30
Left Amygdala	2.616	57	62	30

Right Amygdala	2.591	37	58	30
Left Accumbens	5.781	24	32	56
Right Accumbens	3.367	40	73	34
Brainstem	4.920	52	50	30
Left Lateral Ventricle	5.661	47	62	40
Right Lateral Ventricle	4.109	43	62	41
Left cerebellum	5.710	45	31	19
Right cerebellum	5.700	23	30	14

Table 2. From left to right: brain regions (from the Harvard-Subcortical atlas) that shows positive correlations between the resting state BOLD signal and the SAI; Z-score; X, Y, Z voxel coordinates in the standard MNI 2 mm space.

**Brain Regions**	**Z score**	**X***	**Y***	**Z***
Frontal Lobe				
L-Frontal Pole	5.331	58	93	40
R-Frontal Pole	4.035	38	96	37
L-Superior Frontal Gyrus	3.979	57	73	58
R-Superior Frontal Gyrus	3.981	33	72	59
L-Middle Frontal Gyrus	3.982	58	73	59
R-Middle Frontal Gyrus	3.970	32	74	57
L-Frontal Medial Cortex	3.982	48	87	31
R-Frontal Medial Cortex	3.971	40	89	33
L-Juxtapositional Lobule Cortex	3.300	49	57	62
R-Juxtapositional Lobule Cortex	2.560	43	63	62
L-Paracingulate Gyrus	5.331	49	89	34
R-Paracingulate Gyrus	4.621	42	87	36
L-Cingulate Gyrus, anterior division	4.671	50	85	41
R-Cingulate Gyrus, anterior division	3.943	43	58	51
L-Cingulate Gyrus, posterior division	4.671	47	39	45
R-Cingulate Gyrus, posterior division	5.341	41	42	39
L-Frontal Orbital Cortex	2.549	51	67	27
R-Frontal Orbital Cortex	3.988	38	68	29
L-Precentral Gyrus	4.671	66	57	66
R-Precentral Gyrus	4.625	25	54	70

Parietal Lobe				
L-Postcentral Gyrus	4.012	74	58	52
R-Postcentral Gyrus	4.677	42	45	74
L-Superior Parietal Lobule	3.253	61	35	56
R-Superior Parietal Lobule	2.609	30	41	68
L-Supramarginal Gyrus anterior division	2.564	67	48	54
R-Supramarginal Gyrus anterior division	2.534	12	52	46
L-Supramarginal Gyrus posterior division	2.634	73	42	43
L-Angular Gyrus	3.324	66	32	49
R-Angular Gyrus	3.268	24	35	48
L-Precuneous Cortex	5.350	48	27	51
R-Precuneous Cortex	4.675	43	29	57
L-Central Opercular Cortex	3.981	71	58	43
R-Central Opercular Cortex	3.998	26	57	47
L-Parietal Operculum Cortex	3.269	66	43	46
R-Parietal Operculum Cortex	3.216	17	49	46
Temporal Lobe				
L-Superior Temporal Gyrus anterior division	2.525	72	61	29
L-Superior Temporal Gyrus posterior division	3.222	74	58	31
R-Superior Temporal Gyrus posterior division	3.265	12	51	43
L-Middle Temporal Gyrus anterior division	3.295	75	59	26
L-Middle Temporal Gyrus posterior division	3.290	75	58	26
L-Inferior Temporal Gyrus posterior division	2.574	72	56	22
L-Parahippocampal Gyrus anterior division	2.577	55	52	26
L-Parahippocampal Gyrus posterior division	3.912	54	49	30
R-Parahippocampal Gyrus posterior division	3.916	37	47	32
L-Heschls Gyrus	3.272	67	54	38
R-Heschls Gyrus	3.306	22	53	40
L-Planum Temporale	3.261	76	56	39
R-Planum Temporale	4.008	28	48	44
L-Planum Polare	2.576	68	54	36
R-Planum Polare	2.569	25	52	36
L-Temporal Fusiform Cortex, posterior division	3.196	61	46	21
R-Temporal Fusiform Cortex, posterior division	2.488	32	48	23

L-Temporal Occipital Fusiform Cortex	3.213	61	36	26
R-Temporal Occipital Fusiform Cortex	3.218	30	32	27
L-Subcallosal Cortex	3.255	47	69	34
R-Subcallosal Cortex	3.281	44	72	35
Occipital Lobe				
L-Occipital Fusiform Gyrus	3.994	57	18	27
R-Occipital Fusiform Gyrus	3.301	30	19	28
L-Lateral Occipital Cortex superior division	5.395	63	25	58
R-Lateral Occipital Cortex superior division	4.702	20	29	52
L-Lateral Occipital Cortex inferior division	4.007	60	18	34
R-Lateral Occipital Cortex inferior division	3.989	29	18	28
L-Cuneal Cortex	5.285	45	25	51
R-Cuneal Cortex	4.649	43	24	54
L-Lingual Gyrus	5.307	48	20	27
R-Lingual Gyrus	4.670	43	22	31
L-Intracalcarine Cortex	3.907	47	31	41
R-Intracalcarine Cortex	4.641	41	32	43
L-Supracalcarine Cortex	3.932	49	30	44
R-Supracalcarine Cortex	3.883	44	21	38
L-Occipital Pole	4.019	58	17	28
R-Occipital Pole	4.018	38	13	38
Insula				
L-Insular Cortex	3.944	63	59	41
R-Insular Cortex	3.953	29	50	44

Table 3. From left to right: brain regions (from the Harvard-Cortical atlas) that shows negative correlations between the resting state BOLD signal and the PAI; Z-score; X, Y, Z voxel coordinates in the standard MNI 2 mm space

**Brain Regions**	**Z score**	**X***	**Y***	**Z***
Left Thalamus	5.321	46	61	40
Right Thalamus	4.574	43	51	40
Left Caudate	3.971	53	57	47
Right Caudate	3.990	36	63	48
Left Putamen	3.309	59	55	41

Right Putamen	3.962	37	66	31
Left Accumbens	5.341	41	42	39
Right Accumbens	2.563	39	71	34
Left Hippocampus	5.256	56	52	30
Right Hippocampus	3.959	33	54	28
Left Amygdala	3.900	54	62	29
Right Amygdala	3.961	32	63	30
Brainstem	4.567	51	48	32
Left Lateral Ventricle	4.623	47	62	40
Right Lateral Ventricle	3.874	41	70	42
Left cerebellum	5.307	48	20	27
Right cerebellum	5.244	43	41	38

Table 4. From left to right: brain regions (from the Harvard-Subcortical atlas) that shows negative correlations between the resting state BOLD signal and the SAI; Z-score; X, Y, Z voxel coordinates in the standard MNI 2 mm space.

**Brain Regions**
Frontal Lobe
L-Frontal Pole
R-Frontal Pole
L-Superior Frontal Gyrus
R-Superior Frontal Gyrus
L-Middle Frontal Gyrus
R-Middle Frontal Gyrus
L-Frontal Orbital Cortex
R-Frontal Orbital Cortex
L-Frontal Medial Cortex
R-Frontal Medial Cortex
L-Paracingulate Gyrus
R-Paracingulate Gyrus
L-Cingulate Gyrus, anterior division
R-Cingulate Gyrus, anterior division
L-Cingulate Gyrus, posterior division
R-Cingulate Gyrus, posterior division
L-Precentral Gyrus
R-Precentral Gyrus
L-Juxtapositional Lobule Cortex
R-Juxtapositional Lobule Cortex
Parietal Lobe
L-Postcentral Gyrus
R-Postcentral Gyrus
L-Superior Parietal Lobule
L-Supramarginal Gyrus posterior division
L-Angular Gyrus
R-Angular Gyrus
L-Precuneous Cortex
R-Precuneous Cortex
L-Central Opercular Cortex
R-Central Opercular Cortex
L-Parietal Operculum Cortex
R-Parietal Operculum Cortex

Temporal Lobe
L-Superior Temporal Gyrus anterior division
L-Superior Temporal Gyrus posterior division
R-Superior Temporal Gyrus posterior division
L-Middle Temporal Gyrus anterior division
L-Middle Temporal Gyrus posterior division
L-Inferior Temporal Gyrus posterior division
R-Inferior Temporal Gyrus temporooccipital
L-Inferior Temporal Gyrus temporooccipital
L-Parahippocampal Gyrus anterior division
L-Parahippocampal Gyrus posterior division
R-Parahippocampal Gyrus posterior division
L-Temporal Fusiform Cortex, posterior division
R-Temporal Fusiform Cortex, posterior division
L-Temporal Occipital Fusiform Cortex
R-Temporal Occipital Fusiform Cortex
L-Heschls Gyrus
R-Heschls Gyrus
L-Planum Temporale
R-Planum Temporale
L-Planum Polare
L-Subcallosal Cortex
R-Subcallosal Cortex
Occipital Lobe
L-Occipital Fusiform Gyrus
R-Occipital Fusiform Gyrus
L-Lateral Occipital Cortex superior division
R-Lateral Occipital Cortex superior division
L-Lateral Occipital Cortex inferior division
R-Lateral Occipital Cortex inferior division
L-Cuneal Cortex
R-Cuneal Cortex
L-Lingual Gyrus
R-Lingual Gyrus

L-Intracalcarine Cortex
R-Intracalcarine Cortex
L-Supracalcarine Cortex
R-Supracalcarine Cortex
R-Occipital Pole
L-Occipital Pole
Insula
L-Insular Cortex
R-Insular Cortex

Table 5. Cortical brain regions (from the Harvard-Cortical atlas) that shows positive correlations between the resting state BOLD signal and the SAI and negative correlations between the resting state BOLD signal and the PAI in the standard MNI 2 mm space

**Brain Regions**
Left Thalamus
Right Thalamus
Left Caudate
Right Caudate
Left Putamen
Right Putamen
Left Hippocampus
Right Hippocampus
Left Amygdala
Right Amygdala
Left Accumbens
Right Accumbens
Brainstem
Left Lateral Ventricle
Right Lateral Ventricle
Left cerebellum
Right cerebellum

Table 6. Sub-cortical brain regions and cerebellum (from the Harvard-Subcortical atlas) that shows positive correlations between the resting state BOLD signal and the SAI and negative correlations between the resting state BOLD signal and the PAI in the standard MNI 2 mm space.

**Brain Regions**
Parietal Lobe
R-Supramarginal Gyrus posterior division
Temporal Lobe
R-Middle Temporal Gyrus posterior division
L-Inferior Temporal Gyrus posterior division
R-Inferior Temporal Gyrus posterior division
R-Parahippocampal Gyrus anterior division
Sub-cortical
Left Pallidum
Right Pallidum

Table 7. Cortical brain regions (from the Harvard-Cortical atlas) that shows only positive correlations between the resting state BOLD signal and the SAI in the standard MNI 2 mm space

**Brain Regions**
Parietal Lobe
R-Superior Parietal Lobule
L-Supramarginal Gyrus anterior division
R-Supramarginal Gyrus anterior division

Table 8. Cortical brain regions (from the Harvard-Cortical atlas) that shows only negative correlations between the resting state BOLD signal and the PAI in the standard MNI 2 mm space
